# Psychosocial Correlates of Frailty in Women with HIV in the United States

**DOI:** 10.1093/geroni/igaf122.941

**Published:** 2025-12-31

**Authors:** Anna Rubtsova, Deborah Gustafson, Michael Plankey, Anajali Sharma, Maggie Wang, C Christina Mehta, Andrew Edmonds, Gina Wingood

**Affiliations:** Emory University, Atlanta, Georgia, United States; SUNY Downstate Medical School, Brooklyn, New York, United States; Georgetown University, Washington, District of Columbia, United States; Albert Einstein College of Medicine, Bronx, New York, United States; Emory University, Atlanta, Georgia, United States; Emory University, Atlanta, Georgia, United States; The University of North Carolina at Chapel Hill, Chapel Hill, North Carolina, United States; Columbia University Mailman School of Public Health, New York, New York, United States

## Abstract

Untreated or poorly controlled HIV is associated with frailty, while the use of antiretroviral therapy is protective. Independent of HIV-related factors, whether psychosocial factors are associated with frailty among women with HIV (WWH) has been underexplored. We examined associations of frailty with social support, loneliness, and stress among WWH aged >40 years enrolled in the Women’s Interagency HIV Study (WIHS). We included the latest frailty assessment collected in 2017-2019. Frailty was defined via the Fried Frailty Phenotype as meeting ≥3 of 5 criteria: 1) slow walking speed (4-meter timed gait assessment), 2) reduced grip strength (measured by a dynamometer), 3) low physical activity (self-report), 4) exhaustion (self-report), and 5) unintentional weight loss (self-report). Psychosocial assessments were measured with validated scales (R-UCLA Loneliness, MMOS-SS, PSS-10) and collected in 2015-2017. Separate multivariable logistic regression models assessed for association with each psychosocial measure, adjusting for variables earlier found to be associated with frailty in the WIHS: age, income, obesity, history of cancer, and history of tuberculosis. Our sample included 1250 WWH: mean age 53.4 (SD = 7.59) years, 72% Black, 62% with an annual household income of USD<18,000. Frailty was significantly associated with loneliness (aOR=1.24, 95% CI = 1.11-1.37), perceived stress (aOR=1.04, 95% CI = 1.01-1.06), and emotional social support (aOR=0.99, 95% CI = 0.985 -0.998), but not with instrumental social support (aOR=0.996, 95% CI = 0.989-1.002). These findings suggest that loneliness, perceived stress, and emotional social support may be important factors worth further investigation. Future longitudinal research is needed to examine the relationships between psychosocial factors and frailty among WWH.

